# High-Throughput Video Processing of Heart Rate Responses in Multiple Wild-type Embryonic Zebrafish per Imaging Field

**DOI:** 10.1038/s41598-018-35949-5

**Published:** 2019-01-15

**Authors:** W. Kyle Martin, Alan H. Tennant, Rory B. Conolly, Katya Prince, Joey S Stevens, David M. DeMarini, Brandi L. Martin, Leslie C. Thompson, M. Ian Gilmour, Wayne E. Cascio, Michael D. Hays, Mehdi S. Hazari, Stephanie Padilla, Aimen K. Farraj

**Affiliations:** 1 Curriculum in Toxicology and Environmental Medicine, University of North Carolina at Chapel Hill, Chapel Hill, NC USA; 2 National Health and Environmental Effects Research Laboratory, US Environmental Protection Agency, Research Triangle Park, NC USA; 3 Prince Consulting, LLC, Durham, NC USA; 4 Oak Ridge Institute for Science and Education, Oak Ridge, TN USA; 5 National Risk Management Research Laboratory, US Environmental Protection Agency, Research Triangle Park, NC USA

## Abstract

Heart rate assays in wild-type zebrafish embryos have been limited to analysis of one embryo per video/imaging field. Here we present for the first time a platform for high-throughput derivation of heart rate from multiple zebrafish (Danio rerio) embryos per imaging field, which is capable of quickly processing thousands of videos and ideal for multi-well platforms with multiple fish/well. This approach relies on use of 2-day post fertilization wild-type embryos, and uses only bright-field imaging, circumventing requirement for anesthesia or restraint, costly software/hardware, or fluorescently-labeled animals. Our original scripts (1) locate the heart and record pixel intensity fluctuations generated by each cardiac cycle using a robust image processing routine, and (2) process intensity data to derive heart rate. To demonstrate assay utility, we exposed embryos to the drugs epinephrine and clonidine, which increased or decreased heart rate, respectively. Exposure to organic extracts of air pollution-derived particulate matter, including diesel or biodiesel exhausts, or wood smoke, all complex environmental mixtures, decreased heart rate to varying degrees. Comparison against an established lower-throughput method indicated robust assay fidelity. As all code and executable files are publicly available, this approach may expedite cardiotoxicity screening of compounds as diverse as small molecule drugs and complex chemical mixtures.

## Introduction

Cardiovascular disease is the foremost cause of premature death worldwide^1^, and its incidence and progression are influenced by exposure to drugs, chemicals, and environmental contaminants (e.g., dioxins and polycyclic aromatic compounds), most of which have not been characterized thoroughly for specific phenotypes and/or toxicity potential^2–6^. This need has remained unmet due largely to reliance on protracted and costly mammalian *in vivo* approaches, and *in vitro* methods, which often fail to replicate the sophisticated multi-organ integration in an animal. The zebrafish (*Danio rerio*) is a vertebrate *in vivo* model and a higher throughput alternative to mammalian models. Zebrafish have a high degree of functional conservation with humans, including metabolic processes^7,8^, a sequenced genome with extensive parity to humans, expression of proteins that are orthologous to those known to be involved in over 80% of human diseases^9^, and similarities in cardiac conduction and ion channel composition^10^ resulting in heart rate responses that are predictive of myocardial membrane activity in humans^11^. Moreover, zebrafish embryo skin is permeable to small molecules permitting exposure to an array of drugs and/or toxicants^11–14^, some of which have been linked to changes in expression of human gene orthologues^15^.

Zebrafish have a functional two-chambered heart within the first two days of life^16^, and their transparent skin during early embryonic development allows for unimpeded visualization of the heart using light microscopy, making heart rate a widely measured endpoint in embryonic zebrafish. Current heart rate assays are burdened by some combination of attributes that limit reliability, throughput, and/or transferability to other researchers. These limitations include the use of transgenic lines that express fluorescent proteins in cardiac tissue requiring fluorescence microscopy for visualization^12,17^, the use of costly software and video microscopy systems or robotics, and/or the use of software-hardware combinations that are not commonplace or transferable^18–20^. Additionally, most available methods^17,21,22^, have the capacity to process only one embryo/video at a time.

The main objective of this study was to develop a distributable, simplified, and automated video processing platform that facilitates analysis of single videos or entire directories of videos to measure heart rate of multiple zebrafish embryos per imaging field. Furthermore, we did so in a cost-effective way using only bright field imaging and without the use of anesthesia and/or restraint, addressing limitations of previously published work. To achieve this, 10 second (s), high-resolution, low-magnification video recordings of each well of a 96-well plate, with each well housing two embryos, were obtained using commercially available software. Video files of each well were then processed to acquire heart rate in two steps: (1) use of FisHRateZ, an original algorithm, that capitalizes on the anatomical features of the embryo to automatically locate the heart and derive average pixel intensity vs. time data generated from the cardiac cycle of each embryo, and (2) use of a custom fast Fourier Transform script written and run in open-source software that enables batch derivation of heart rate from FisHRateZ data. To examine the utility of this approach, we first assessed responses to two drugs with well characterized pharmacological effects in humans and known influences on heart rate in zebrafish^11^. Then, given the linkage of respirable particulate matter (PM), a complex mixture of environmental contaminants with adverse cardiovascular outcomes^2,23–25^, we also compared the effects of organic extracts from different sources of air pollution-derived PM, including petroleum diesel and soy biodiesel exhausts, and red oak biomass smoke.

## Methods and Materials

### Image Capture

Black, 96-well, flat-bottom, polystyrene microplates (Greiner Bio-One North America Inc., Monroe, NC) were covered with a one mm thick light scattering panel (Nikon SW-12 Diffuser Panel, Nikon Instruments, Melville, NY) employed to cast a consistent field of illumination throughout the well. Images were captured using an Andor Zyla 4.2 sCMOS (Andor Technologies, Belfast, NI) camera mounted to a Nikon Ti microscope (Nikon Instruments, Melville, NY) equipped with a 2x objective. This camera enabled capture of the entire well within a single field of view, allowing all embryos within the well to be visible simultaneously. NIS-Elements (version 4.13, Nikon Instruments, Melville, NY) was used to control the camera and the microscope with a motorized stage (Prior Scientific, Rockland, MD). A sequence of video captures was programmed that acquired videos of wells in a “serpentine” pattern, moving across successive rows of wells in alternating directions from the top to the bottom of the plate. As each well was moved into position, light was applied from a halogen source for 20 s. The first 10 s of illumination served to acclimate the animals to the brightened lighting conditions. Following the first 10 s of illumination, images of stationary embryos were captured at 16.5 frames/s for 10 s and saved as time-sequence image stacks. The camera was configured to capture images at 11 bits/pixel with a resolution of four megapixels and an actual spatial resolution of 3.24 µm/pixel. Following the programmed illumination time, light was interrupted via microprocessor-controlled shutter (Sutter Instruments, Novato, CA), and the motorized stage moved the next well into view and the process was repeated until completion of the plate.

### Automated Imaging Analysis of Video Files

A two-step approach was developed that derived heart rate in wild-type embryonic zebrafish by capitalizing on the changes in pixel intensity resulting from the contraction and relaxation of the heart during each heartbeat. The first step involved creation of pixel intensity profiles using automated image analysis of 10 s video files generated for each entire well containing two embryos/well. Given that heart tissue is more opaque than static background, contraction of the heart increased average brightness within the prescribed region of interest (ROI); conversely, relaxation decreased average brightness. The algorithm recorded these regular oscillations in brightness as changes in average pixel intensity over time. Next, the average pixel intensity vs. time data were converted to a frequency spectrum using fast Fourier Transform (FFT), a method applied previously to determine heart rate in fluorescently-labeled embryos^12^. The dominant frequency corresponded to heart rate.

**Figure 1:**
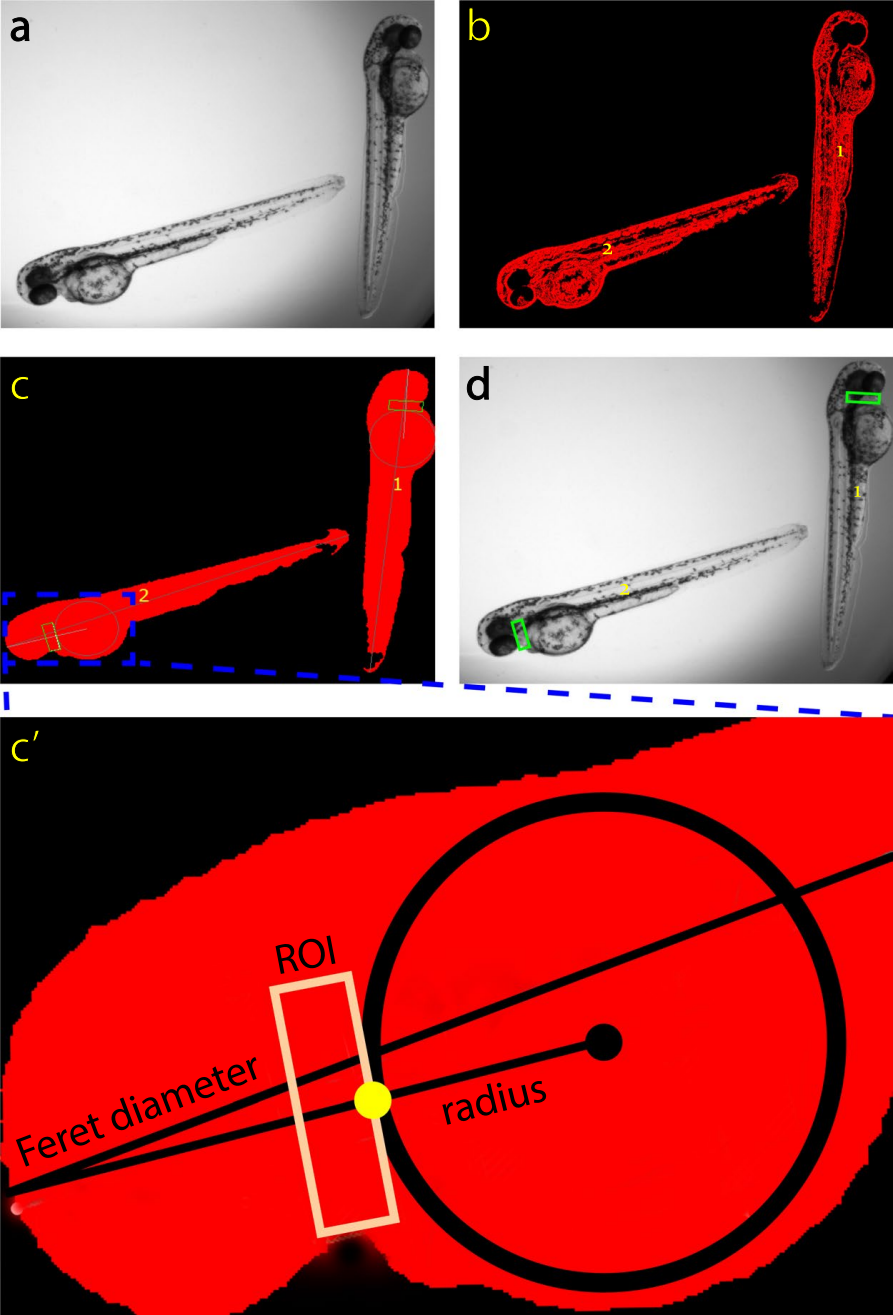
Image processing using FisHRateZ. (**a**) original image pulled from the 10-s video file. (**b**) FisHRateZ identifies embryos and removes all other particles (i.e., lint or dust) in wells. (**c**) The shape of the embryo allows for geometric determination of heart location for ROI placement. c’) inset of heart region of embryo shows: a Feret diameter, which is the longest line that can be drawn lengthwise across the embryo, a circle superimposed over yolk sac, a second line passing through the circle, and a region of interest rectangle (ROI) as described in the Methods and Materials section. (**d**) ROI boxes are superimposed over the original image and applied to each frame of the video for pixel intensity versus time plotting.

Average pixel intensity data were generated automatically using an original algorithm that was written in LabVIEW (National Instruments Inc., Austin, TX), but requires only a Vision Deployment Module Run-Time License for operation. LabVIEW software is required to view and edit the source code. Upon loading the video file(s) (Fig. 1a), the algorithm: (a) automatically detected each embryo within each well and assigned a unique identifier to each embryo (i.e., “1” or “2”); (b) automatically found the heart region of each embryo and overlaid a rectangular region of interest (ROI) containing both the ventricle of the heart and a section of static background that restricted pixel intensity measurement to this region, effectively excluding measurement of pixel intensity oscillations caused by non-heart tissue or artifacts; and (c) calculated the mean pixel intensity data for the duration of the video and exported the data in a text file. These automated steps are described in greater detail below:

#### Identification and Isolation of Each Embryo

Digitally isolating the embryos required a series of sequential functions that included edge detection and masking (which limited processing to items within the well), thresholding (which isolated the embryos from background), and digital removal of non-embryo particulates (e.g., lint, dust, etc.). At the end of this step, the only items that were identified in the well were embryos (Fig. 1b).

#### Locating the Heart

Next, the unique shape of the embryo was utilized to identify the position for placement of the ROI. The natural position of the heart, the site for ROI placement, is on the ventral surface of the zebrafish embryo, rostral to the yolk sac, with the ventricle being dorsal and rostral to the atrium (Supplemental Fig. 1). To identify the position for ROI placement, the algorithm first identified the yolk sac, which corresponded to the largest circular object within the embryo, and used it as a landmark. To ensure placement of the ROI rostral to the yolk sac, the algorithm then determined the embryos’ Feret diameter, which is a measurement used in estimating the size of non-uniform and randomly oriented objects^26^, and was the longest line that could be placed lengthwise along the embryo. Given that the yolk sac is rostral to the midline of the long axis of the animal, the circle that overlaid the yolk sac divided the Feret diameter into shorter and longer segments, with the shorter segment corresponding to the head region. The shorter section of the Feret diameter was used as the guidepost for placement of the ROI rostral to the yolk sac. To place the ROI closer to the ventral side of the embryo such that the ROI overlapped the ventricle, a line was drawn from the center of the circle to the end of the short segment of the Feret diameter. The algorithm then identified the point at which this line intersected the perimeter of the circle, and the ROI was placed tangent to the circle at this point (Fig. 1c and c’). The use of this intersection point allowed for placement of ROIs irrespective of embryo orientation in the *x* − *y* plane. The generated ROI was one radius of the circle in width and 1/3 radius in height and primarily covered ventricular heart muscle. These ROI scaling factors were the default settings and are adjustable.

#### Application of ROI to Video Files

The ROI rectangles were superimposed over the embryos for all frames in the video (Fig. 1d) followed by simultaneous calculation of the mean pixel intensity value for each ROI. The algorithm exported results in a tab delimited text file.

For quality control, a still image from the video file that showed all embryos with their ROIs superimposed was saved automatically to a user-specified directory. This allowed quick visual confirmation that the ROI was placed correctly. In addition, the software generated a pixel intensity vs. time plot which provided an opportunity to check for unexpected pixel intensity patterns that may have arisen due to embryo movement. This algorithm was run on a Dell T3600 equipped with an Intel Xeon e5-1620 processor running at 3.6 GHz, and an AMD FirePro W5000 graphics card.

### Derivation of Heart Rate from Pixel Intensity Profile

The procedure described above identified the ROI and provided about 165 data points captured during the 10 s video. An original script run in the open-source software GNU Octave (https://www.gnu.org/software/octave/#install) was developed for processing of these data to derive the heart rate. Two steps were involved in this processing. Initially, we corrected for baseline drift that occurred occasionally while the video was being recorded. Then, without further intervention by the user, a fast Fourier Transform was applied to obtain frequency values from the baseline corrected data. The dominant frequency was the heart rate (Supplemental Fig. 2).

#### Correction for baseline drift

Baseline drift resulted from an overall increase in intensity of the sample. Due to the sensitivity of the camera, small changes in image illuminations could be reflected as a change in image pixel intensity that were not reflective of the beating heart.

The baseline drift correction procedure consisted of the following steps:

1. The ROI data, about 165 data points collected during the 10 s video, were divided into 10 equal segments. Thus, each segment contained about 1 s of data.
2. The average value of the ROI data in each of the 10 s segments was calculated.
3. For segments 2–10, the difference between the segment average value and the average value of segment 1 was calculated (i.e., segment “n” – segment 1). For each segment, this difference could be either positive or negative. For example, if the average value of the ROI data for segment 2 was larger than the corresponding average for segment 1, then the difference would be positive, and vice versa.
4. For segments 2–10, the ROI data were adjusted by subtracting the differences as calculated in Step 3 immediately above. Therefore, if the average value of segment 2 was greater than the average value for segment 1, the ROI values for segment 2 were all decreased, and vice versa.
5. With a monotonic baseline drift that was either positive or negative, this method applied a progressively larger correction as segment ROI values diverge from the average value of the ROI data for the first segment. Division of the ROI data into 10 s segments proved sufficient to produce a visibly substantial reduction in baseline drift.

#### Fourier Transform

The Fourier Transform function was used to identify the dominant frequency of the ROI data. This procedure included the following steps:

1. The differences between the value of each data point and the mean of all the data points were calculated.
2. The time step between data points was calculated.
3. The data sampling frequency (radians/s) was calculated by 2π/time step.
4. The frequency axis was built from 0 to the Nyqist frequency, which is the sampling frequency/2.
5. The *fft* function in GNU Octave was used to calculate the discrete Fourier transform of the data as modified in Step 1 immediately above.
6. The Fourier Transform was multiplied by the time step.
7. The preceding steps provided the heart rate.

**Figure 2:**
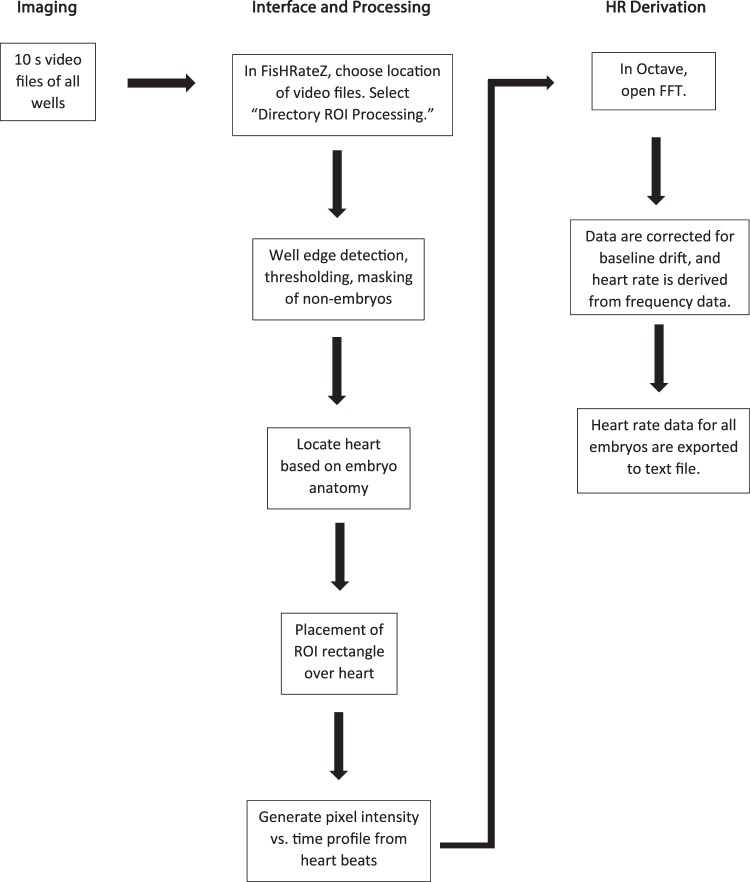
Workflow of heart rate derivation using FisHRateZ and original FFT.

Biologically plausible lower and upper bounds for zebrafish heart rate were specified from a survey of the literature. The accompanying supplementary video provides a tutorial for the method presented here, and Fig. 2 provides a work flow from imaging to heart rate derivation.

### Comparison of Heart Rate Data from Automated vs. Manual ROI Placement

After imaging, a researcher blinded to the exposure groups manually ascribed an ROI over the ventricle for each embryonic heart that was captured by the automated algorithm across 10 experiments (two experiments each for five of the exposure drugs or mixtures) selected at random. A macro in NIS-Elements allowed for this manually placed ROI to be applied to all frames in a video in a single step. The output file was similar to the output of the automated algorithm, measuring heart contraction patterns as changes in average pixel intensity vs. time. An FFT run in Octave provided heart rate in a text file. The manually-derived heart rate values were compared to the output of the algorithm using linear regression, Pearson’s correlation, and Bland-Altman methods comparison.

### Chemicals and Drugs

Epinephrine hydrochloride (HCl; racemic; ≥90% purity), clonidine HCl (≥99.9% purity), and dimethyl sulfoxide (DMSO; ≥99.9% purity) were purchased from Sigma Aldrich (St. Louis, MO). All salt components of 10% Hanks’ Balanced Salt Solution, hereafter referred to as “HBSS”, (13.7 mM NaCl, 0.54 mM KCl, 25 µM Na_2_HPO_4_, 44 µM KH_2_PO_4_, 130 µM CaCl_2_, 100 µM MgSO_4_, and 420 µM NaHCO_3_) were obtained from Sigma-Aldrich and combined in deionized water. Organic extracts of PM_2.5_ (particulate matter with an aerodynamic diameter ≤2.5 µm) were generated in-house as described previously: compressor-generated diesel exhaust particulate (**CDEP**)^27^, petroleum diesel (0% biodiesel, **B0**), soy biodiesel (100% biodiesel, **B100**), and a 1:1 blend of B100 and B0 (**B50**)^28^. Extracts of a condensate of a high temperature combustion of red oak (*Q. rubra*; **RO**) were generated and prepared as described previously^29^. Extracts contained the solvent-extractable organic material (EOM) from each combustion which was composed of many of the leachable elements of air pollution-derived PM. Extracts and condensates were suspended in DMSO, and the drugs were in HBSS. All drugs and chemicals were stored at 4 °C and protected from light except for HBSS, which was stored at room temperature for no more than one week.

### Aquaculture and Animal Husbandry

All studies were carried out in accordance with the guidelines of, and approved by, the Institutional Animal Care and Use Committee at the U.S. EPA’s National Health and Environmental Effects Research Laboratory. The fish were housed in an Association for Assessment and Accreditation of Laboratory Animal Care-approved animal facility with a 14:10 h light:dark cycle with lights on at 08:30 h. Embryos were reared and bred as described previously^30^. Briefly, breeding utilized an in-house strain of zebrafish (*D. rerio*; undefined, outbred stock originally obtained from Aquatic Research Organisms, Hampton, NH and EkkWill Waterlife Resources, Ruskin, FL) that were bred annually to wild-type fish to increase genetic diversity. Male and female adults were housed together at ~8/liter, (Aquaneering Inc, San Diego, CA) with a water temperature of 28 °C. Adult fish (80–100) were selected to form spawning groups in 15-liter static tanks the afternoon before collection. Embryos were collected between 8:30 and 9:00 h the following morning. All embryos were gathered from the breeder tank and placed in a beaker and into a 28 °C water bath and washed as described previously^31^. Embryos were reared in 10% HBSS.

### Sample size analysis

Sample size analysis was conducted for a test of the hypothesis that exposure to PM extracts in embryonic zebrafish would change heart rate by 15 beats/minute (bpm), which reflected roughly a 10% change. This value was derived from observations of data from the positive controls epinephrine HCl and clonidine HCl. Vehicle control groups (0.4% DMSO) from these data sets yielded a heart rate standard deviation (SD) of approximately 12 bpm. The vehicle control SD from these control experiments were used to calculate the effect size (d) and effect size index (f) for sample size calculations.

$$\begin{array}{rcl}(d) & = & \frac{expected\,effect\,range}{SD}=\frac{15}{12}=d=1.25\\ (f) & = & 0.5\,(d)=0.5(1.25)=0.63\end{array}$$

We used R Studio software to perform sample size calculations with the *pwr* package (https://cran.r-project.org/web/packages/pwr/pwr.pdf) and *pwr.anova.test* command. The number of experimental groups was one vehicle control group and four PM extract concentrations (k = 5). Standard power and p values were used: power = 0.8, sig. level = 0.05. We set f = 0.63, which produced an n = 7.03, so the raw calculated necessary sample size was taken as 8. An additional sample was added to the rounded sample size for potential unaccounted error, and a loss rate allowance of 25% was added bringing the final sample size to 12 wells/group for the study. For most plates in the study, extra wells were used for vehicle and/or highest concentrations of extract samples.

### Experimental Design for Drug and PM Extract Screening

The embryos developed in HBSS until 4–6 hours post-fertilization (hpf) when they were selected for experimental purposes. The embryos were placed into a polystyrene Petri dish (Fisher Scientific, Waltham, MA) with HBSS and then into an incubator (26 °C) overnight. At approximately 24 hpf, the embryos were dechorionated manually using ultrafine tip forceps (this is required because the folded orientation of the embryo within the chorion does not permit imaging of the heart, and embryos do not emerge from the chorion until ~72 hpf) and placed two/well in a 96-well plate with 250 µl HBSS per well, returned to the incubator and housed overnight.

At approximately 48 hpf, the embryos were exposed to epinephrine HCl (10, 40, or 80 µg/ml), clonidine HCl (3, 10, 30, or 100 µg/ml), CDEP, B0, B50, B100, or RO (0.1, 1, 10, or 40 µg EOM/ml for all fuel sources; PM extract stock solutions were vortexed thoroughly), or a vehicle control of DMSO (0.4% or 0.8%) (Supplemental Table 1). The drug concentrations were similar to those from previous publications^11,12^ and the extract concentrations were chosen to provide a wide exposure concentration range. Only three concentrations of epinephrine HCl were tested because of increased locomotor activity in the wells, decreasing capture rate. The pH of the extracts at the highest exposure concentration (40 μg/ml) was 7.2–7.8 (Table 1), within the range considered safe for zebrafish rearing^32^. Five minutes were allowed for dispersion of extract before 150 µl was removed to achieve optimal optical clarity for microscopy. Dosing and removal of media took 10 minutes. The embryos were returned to the incubator for five hours, then placed on the stage of a Nikon Ti microscope for 20–30 minutes to acclimate at room temperature. The total time of exposure before imaging was 5.5 hours, which was selected as the exposure duration because 1-hour treatments caused irritant-induced locomotor responses that precluded image captures. A pre-defined exclusionary criterion of ±2-fold the SD from the pilot data control mean was used to exclude animals from control groups; no animals were removed from treated groups unless they were dead or morphologically abnormal by the end of the experiment. Heart rates of all captured embryos in each well were averaged, yielding a well-averaged heart rate. Experiments were run in duplicate on separate days and the data from the two experiments were combined once they were confirmed not to contain statistically different data.

**Table 1:** Chemical characteristics of extracts.

Sample	Extractable Organic Material (EOM; %)	Concentration of PAC (% of EOM)	Total Chemical Profile of EOM Characterized (%)	pH at 40 µg/mL of EOM	Reference
CDEP	23	0.1	0.1	7.3	Mutlu *et al*.^27^
BO	26	0.102	2.97	7.2	Mutlu *et al*.^28^
B50	32	0.075	10.05	7.2	
B100	60	0.016	24.75	7.2	
RO	70	0	4.1	7.8	Kim *et al*.^29^

### Statistics and Data Analysis

All statistics and data analyses were completed in Graphpad Prism 6 or 7 (Graphpad Software, Inc., La Jolla, CA) except for sample size determination which was completed in R Studio. Many data are visualized as boxplots with all data points shown. Box top and bottom delineate the interquartile range, the middle line marks the median, the “ + ” marks the mean, and the whiskers mark the range. Dashed lines carry the control mean value across each panel where boxplots are used to visualize the data. We assessed normality of data distributions with a D’Agostino-Pearson omnibus normality test with significance set at p < 0.05. All groups from epinephrine HCL, CDEP, B0, B100, and RO met this assumption (p > 0.05). The clonidine HCl experimental data contained one group (30 µg/ml) that had a p-value < 0.05 (p = 0.0333), so the data were analyzed with both ANOVA and Tukey’s multiple comparisons as well as a Kruskal-Wallis test with a Dunn’s multiple comparisons (parametric and non-parametric, respectively). The ANOVA produced the most conservative results and the p-values associated with these data represent those derived from the ANOVA. The B50 experimental data produced two groups that were not normally distributed (1 µg/ml [p = 0.0164] and 10 µg/ml [p = 0.0412]). The data were analyzed with both ANOVA and Tukey’s and Kruskal-Wallis and Dunn’s; neither produced significant results. For comparison of control data, all three groups contained normally-distributed data, per previous analysis, and were compared with ANOVA and Tukey’s. Linear regression analysis (r^2^, p-value, and slope) and Pearson’s correlation analysis (r value), respectively, determined goodness of fit and degree of correlation of heart rate data used to compare manual and automated placement of ROI rectangles. A Bland-Altman analysis was performed to quantitatively test the difference between the two methods. The metric ‘bias’ describes the lack of agreement between the two methods (i.e., when the two methods return different results, bias indicates by how much on average they are different), and the 95% limits of agreement delimit the interval in which 95% of all differences between the methods exist^33^. For the extract exposures, we performed linear regressions of the heart rate responses to determine slopes, which were used to indicate the potency of each extract and were expressed as bpm/μg EOM/ml. Slopes of the resulting lines were compared with a one-way, two-tailed ANOVA and a Tukey’s multiple comparisons test. Note: for this analysis, the slopes derived from the linear regression of the concentration-response for each extract were entered into Prism as the mean, the standard error for the slope, also derived from the linear regression data, was entered as the standard error of the mean, and the degree of freedom (df) value + 1 was entered as “n” or group size; the df for all groups in this analysis was 1. These parameters were defined by Graphpad Prism, Inc.^34^. Replicate experiments were compared using one way, two-tailed ANOVA and Tukey’s multiple comparisons test and combined if not different.

## Results

### FisHRateZ capture rate

The capture rate of FisHRateZ was measured in two ways: first, by overall capture of imageable embryos, where each embryo was the biological unit. Imageable embryos were defined as embryos that were alive, morphologically normal, remained stationary for 10 s, and were in focus in the *z* plane (vertically within the water column). FisHRateZ captured 77% of all imageable embryos in less than 50 minutes/experiment. Capture rate was also measured after treating each well of the 96-well plate as the biological unit with capture rate of imageable wells here defined as a well with no dead or abnormal animals, with at least one embryo that stayed stationary and in focus in the *z* plane. FisHRateZ captured 77% of imageable wells. Table 1 shows capture rates across five groups.

### Heart rates derived from automated ROI placement match those derived from manual placement

**Figure 3:**
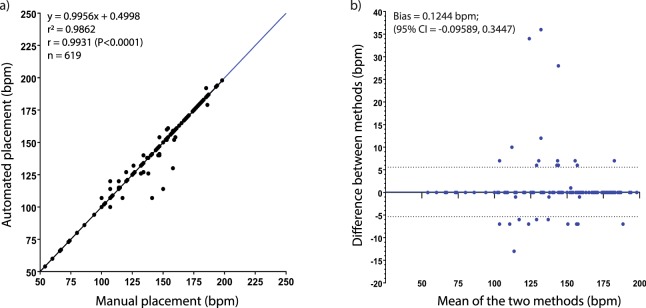
A comparison of heart rates derived from automated placement of Region of Interest match those derived via manual placement. (**a**) Heart rate data from both ROI placement methods were plotted against one another and linear regression derived r^2^, slope and p-value. r value indicates correlation of manual and automatic placement of ROI-derived data of all imaged animals across 10 total experiments. The line of identity (blue) illustrates a hypothetically “perfect” correlation between the two data sets for comparison. (**b**) A Bland-Altman analysis provides quantitative data on the disagreement between the two methods (bias) and the interval within which 95% of all disagreement occurs (95% limits of agreement indicated by dashed lines; −5.346 to 5.594). The x-axis is the average between the heart rates derived from the two methods, and the y-axis is the distance that those two values are from one another (Method A-Method B). A positive value on the y-axis indicates that Method A (manual ROI placement) returned a higher value, and vice versa.

To assess the capability of the algorithm to place ROIs automatically and correctly over the hearts, a researcher blinded to the experimental groups placed ROIs manually over all embryos captured by the algorithm across a collection of 10 experiments selected randomly. Linear regression of plots of algorithm vs. manual ROI placement heart rate data was conducted for these 10 data sets to derive an r^2^ goodness of fit value and slope, and Pearson’s correlation derived an r value. When all the data were combined from 10 experiments, r = 0.9931 (95% CI: 0.9919–0.9941; p < 0.0001) and r^2^ = 0.9862 (Fig. 3a). An identity line (in blue) illustrated a hypothetically perfect correlation (r = 1) between two data sets containing data that should be identical under ideal conditions^35^, and allowed for visual comparison with experimental data. The high r value indicated a strong correlation between the data sets, and high r^2^ values indicated that the values derived from manual placement of ROI were predictive of values derived from automatic placement. Given the strong, positive, linear relationship between the two data sets, a Bland-Altman Methods comparison analysis was run to quantify disagreement between methods. The metric ‘bias,’ used to describe disagreement between the two methods used to derive heart rate, was 0.1244 bpm (95% CI: −0.09589 to 0.3447), which is not a difference we considered to be meaningful. The 95% limits of agreement (−5.346 to 5.594) indicate where 95% of the differences between the two methods can be expected (Fig. 3b). Furthermore, there was no obvious impact of direction of change of heart rate on the bias (i.e., increases or decreases in heart rate did not increase or decrease bias). A frequency distribution of the differences between values derived using the two methods showed that in 94.7% (586/619) of comparisons, the difference was 0 bpm (Supplemental Fig. 3).

### Responses to drugs known to modulate heart rate

**Figure 4:**
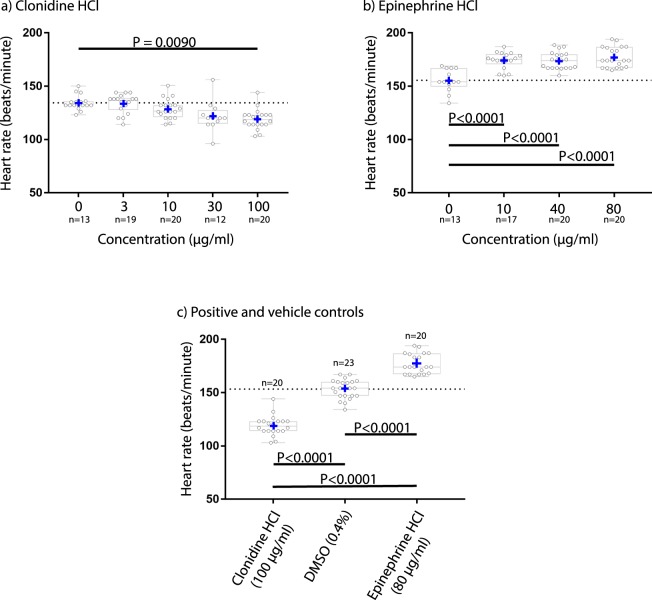
FisHRateZ successfully captured increases and decreases in heart rate. (**a**) clonidine HCl, (**b**) epinephrine HCl, (**c**) Side-by-side comparison of clonidine HCl 100 μg/ml, 0.4% DMSO, and epinephrine HCl 80 μg/ml. Concentration = 0 is 0.4% dimethyl sulfoxide (DMSO) vehicle control. One-way, two-tailed ANOVA and Tukey’s (panels a and c) or Kruskal-Wallis and Dunn’s (panel b; see Methods and Materials).

To demonstrate that FisHRateZ could detect decreases in heart rate, the anti-hypertensive drug clonidine HCl was used to lower heart rate. Clonidine decreased heart rate by 14 ± 9.3 bpm (10.5% decrease) at a concentration of 100 µg/ml (Fig. 4a) but did not at lower concentrations. To assess the capacity of FisHRateZ to measure increased heart rate, the non-selective adrenergic agonist epinephrine HCl was administered. Epinephrine increased heart rate at all concentrations with a maximum increase of 22 ± 9.5 bpm at 80 µg/ml (13.9% increase; Fig. 4b). FisHRateZ accurately and precisely captured increased and decreased heart rates (Fig. 4c). These results are expressed as mean ± SD and have associated p-values of ≤0.0090, which are reported individually in Fig. 4.

### Heart rate responses for embryos treated with PM extracts

**Figure 5:**
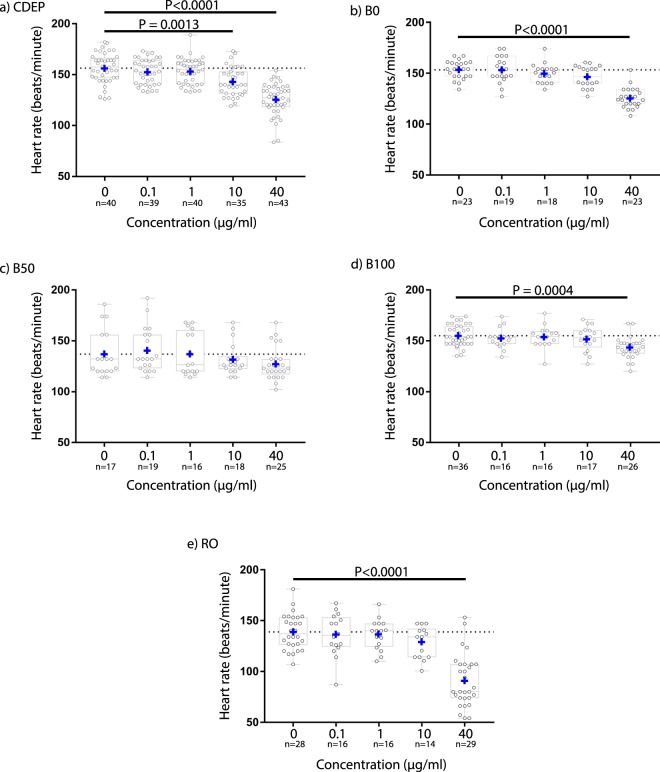
Heart rate responses to five organic extracts of complex mixtures of air pollutants. (**a**,**b**) pure petroleum diesels CDEP and B0. (**c**) B50, 1:1 blend of B0 and pure soy biodiesel. (**d**) B100, pure soy biodiesel. (**e**) RO, flaming red oak wood smoke. One-way, two-tailed ANOVA and Tukey’s.

Zebrafish embryos were treated with organic extracts of combustion byproducts from two petroleum-based diesel fuels generated under different conditions (CDEP and B0), a pure soy biodiesel (B100), a 50/50 blend of B100 and B0 (B50), and emissions from combusted red oak wood (RO) to assess impacts on heart rate. Chemical composition of the extracts is noted in Table 2. CDEP depressed heart rate at 40 µg/ml (31 ± 15.5 bpm; 19.6% decrease) and 10 µg/ml (13 ± 14.2 bpm; 8.5% decrease; Fig. 5a). B0 caused a similar decrease in heart rate at 40 µg/ml (28 ± 9.9 bpm; 18.0% decrease), but there was no effect at 10 µg/ml (Fig. 5b). Intriguingly, B50 did not alter heart rate at any concentration (Fig. 5c). Compared to the petroleum diesel-derived samples, B100 elicited a milder decrease in heart rate of 12 ± 10.6 bpm at 40 µg/ml (7.6% decrease; Fig. 5d). RO precipitated the greatest reduction in heart rate among the PM-derived extracts with a reduction of 48 ± 25.8 bpm at 40 µg/ml (35% decrease; Fig. 5e). These results are expressed as mean ± SD and have associated p-values of ≤0.0013, which are reported individually in Fig. 5.

**Table 2:** Capture rate of FisHRateZ by percentage of embryos and wells captured.

	CDEP	Bo	B50	B100	RO	Total
Capture rate by embryo (%, mean ± SEM)	74 ± 1.2	82.5 ± 7.5	71.5 ± 0.5	86.5 ± 2.5	69 ± 3.0	77
Capture rate by well (%, mean ± SEM)	75 ± 1.5	82.5 ± 7.5	71.5 ± 0.5	87 ± 2	70 ± 3	77

### Potency of PM Extracts

**Figure 6:**
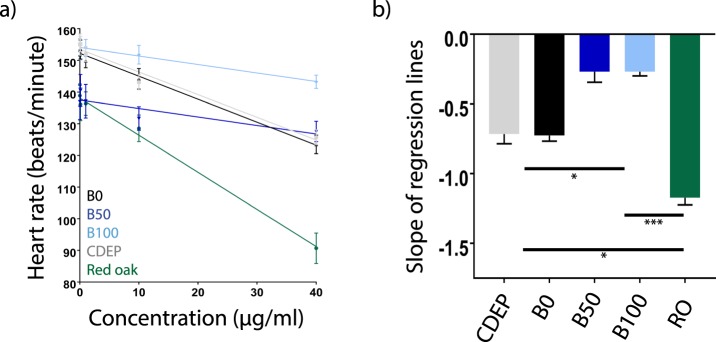
Potency of complex mixtures of air pollutants. (**a**) linear regressions of heart rate responses for RO (green), CDEP (silver), B0 (black), B50 (dark blue), and B100 (light blue). (**b**) Comparison of slopes, which were used as an indicator of the relative potency of the extracts (mean ± SEM). Slope values can be read as Δbpm/µg extractable organic matter/ml. (*p < 0.05, ***p < 0.001). One-way, two-tailed ANOVA and Tukey’s.

The slope of the linear regression derived from the concentration-responses of each extract was used to determine the potency as described previously^27,30^ (Fig. 6a). Statistically, the extracts fell into three groups based on comparison of slope of the regression lines using ordinary one-way, two-tailed ANOVA and Tukey’s multiple comparisons test. B50 and B100 clustered together, as these samples had the least impact on heart rate. The two extracts of diesel exhaust particulate (CDEP and B0) had a greater impact on heart rate and clustered into an intermediate-effect group. RO was the most potent, causing the most pronounced reduction in heart rate (Fig. 6b).

## Discussion

Here we present a high-throughput video-processing platform for heart rate detection in multiple wild-type zebrafish without the use of restraint, anesthesia, or fluorescence, and with source codes and executable files available for immediate download. This platform applied an original, automatic, and robust image processing routine to batch process video files of embryonic zebrafish heart contractions using edge detection, thresholding, and filtering. FisHRateZ automatically (1) detected multiple embryos/well within a 96-well plate, (2) overlaid an ROI on the heart regions of each embryo for pixel intensity measures of heart contraction patterns, and (3) logged unique intensity vs. time data for each embryo for post-processing determination of heart rate. Intensity data were then batch-analyzed using a custom, user-friendly FFT script to yield heart rate. The platform presented here completed accurate and precise video processing of all videos from each 96-well plate in ≤50 minutes across a dynamic heart rate response range, was responsive to changes induced by small molecule drugs and chemical mixtures, and provided data that were used to cluster complex mixtures into distinct groups based on degree of impact on heart rate. Furthermore, because thousands of videos can be processed within hours, FisHRateZ may increase the throughput and reliability of phenotype-based cardiotoxicity screening efforts. Finally, because we make the code publicly available, researchers can alter the software to meet their research needs, including other microplate formats, and examination of adaptability to other small model organisms.

Our platform overcomes several challenges inherent in previously published approaches to heart rate assessment in embryonic zebrafish. FisHRateZ is the first approach to simultaneously detect and place ROIs on multiple wild-type embryos/well, including up to five embryos/well; however, optimization efforts supported two/well as the ideal number. No other published approach reports processing of more than one wild-type embryo/well. Importantly, detection of multiple embryos/well reduced the number of wells lost to embryo movement or poorly focused animals. Additionally, we determined that co-housing of embryos, up to four/well, had no significant impact on heart rate (Supplemental Fig. 4), further supporting the assessment of multiple embryos/well. Our approach also processed videos captured in bright field, bypassing the need for transgenic embryos labeled with fluorescent markers that require fluorescent and/or confocal microscopy^12,17^ which is both costlier and more technically challenging. Moreover, the capacity to process bright field-acquired video images opens the door for assessment of any zebrafish strain, affording researchers with varied interests the flexibility to measure this critical heart health endpoint. Furthermore, Bland-Altman analysis indicated that FisHRateZ was equally capable of detecting increases or decreases in heart rate (50–200 bpm in the present study), suggesting that it is appropriate for use with genetic models in which brady- or tachycardia are observed. By focusing on 2 dpf embryos prior to swim bladder inflation, the requirement for anesthesia or immobilization, both of which have confounding effects on heart rate^36^, is circumvented. Additionally, assessments at this time point are not limited by the requirement to manually position the animal for proper orientation, which was required in previously published studies^19,20,22,37,38^, since the algorithm was capable of precisely placing ROIs over the hearts of embryos that were either laying on their side (lateral) or supine (ventral side up) (Supplemental Fig. 5); orientation of embryos with dorsal side up was not observed. We were also able to successfully capture heart rate without the use of 1-phenyl-2-thiourea, which inhibits pigment development and may confound responses. Furthermore, FisHRateZ locates the heart based on the unique shape of each embryo and ultimately derives heart rate from movement of heart muscle wall, not relying on surrogate measures (e.g., body deformation, pulsatile blood flow, etc.) as described by other recent approaches^22,38–40^, which may be susceptible to movement artifacts or organ malformations that often take place with chemical exposure. FisHRateZ also presents a user-friendly interface, requiring only a simple command to process either single videos or entire directories (Supplemental Fig. 6) and derive data that are handled by a custom FFT that requires only a simple command itself. The algorithm also saves a still image from the video onto which the ROI is superimposed allowing for rapid quality assurance checks that ensure proper placement. FisHRateZ was built in a software platform used routinely in the research community, and the FFT was written in an open-source interface, with source codes and executable file made publicly available to increase utility, and to address data reproducibility.

Epinephrine HCl and clonidine HCl caused increases and decreases in heart rate, respectively, indicating sensitivity of FisHRateZ to capture changes in heart rate across a spectrum of responses, without lesser sensitivity at either low or high heart rate. Importantly, data derived from FisHRateZ were not different from those derived by manual placement of the ROI, which indicated robust assay fidelity. In addition, control animal heart rate responses reported here were well within the range of heart rate responses reported by others^17,41,42^. Furthermore, FisHRateZ detected statistically significant changes in heart rate with high resolution, including a change of only 7.6% in one case, presenting the opportunity to probe subtle phenotypic changes in cardiovascular function.

The data generated using FisHRateZ provided an opportunity to explore potential mechanisms of action addressing the disparity of responses after PM extract exposure. We anticipated that potency (Δbpm/µg EOM/ml) would peak for samples with higher levels of polycyclic aromatic compounds (PAC), which consist of polycyclic aromatic hydrocarbons and their oxy- and nitro- derivatives, and have been directly implicated in the pathogenesis of human cardiovascular disease^43–48^. The pure diesel combustion products of CDEP and B0 were richest in PACs (Table 1) and caused greater depression in heart rate than B100 and B50, consistent with other findings^49^. The diminished responsiveness to B50 exposure is congruous with published work linking blended fuel combustion byproducts with reduced biological impacts when compared to effects caused by emissions from pure petroleum diesel^28,50–52^.

Although non-PAC constituents (e.g., quinones) of the PM extracts may have contributed to the observed responses, PACs are known cardiotoxicants and PM-derived PACs have been linked to PM-induced cytotoxicity and oxidative stress, among other mechanisms^45,53,54^. Aberrant Ca^2+^ handling and cardiac rhythm have been demonstrated with phenanthrene^55^, a PAC present in air pollution-derived PM. Interestingly, the extract from flaming red oak combustion produced the most robust depression of heart rate with a reduction of approximately 35%. This sample was not exceptionally rich in PAC, but was rich in other semi-volatile organic compounds such as methoxyphenols^29^, which tend to be present in large quantity in wood smoke^56^, and are known to interfere with excitation-contraction coupling in heart and vascular tissue, principally through aberrant Ca^2+^ handling^57–60^. These findings are consistent with epidemiological findings and data from experimental mammalian models that indicate that chemical composition of PM air pollution plays a key role in the elicitation of adverse cardiovascular health effects^2,50,51,61–63^. The precise mechanism of action accounting for the observed decreases in heart rate, and the degree to which responses are dependent on pollutant composition remain uncertain.

Some limitations of the present approach must be considered. Although 77% of all imageable embryos were successfully captured, on par with published single embryo/well methods that use robotic systems^19,20^, increases in capture rate would further increase utility. Loss was often attributable to ROI misplacement or non-placement on embryos deemed imageable by the standards outlined in the Methods and Materials section. The precise reason for these errors is unclear, but may relate to turbidity of the media due to chemical treatment, downward positioning of embryos in the *z* plane, or proximity of the embryo to the well edges, which tend to be darker. In addition, the algorithm was typically unable to assign precise ROIs on embryos that overlapped, which increased in likelihood with three or more embryos/well, and occurred only rarely with two embryos/well. In some instances, the ROI also overlapped some atrial tissue, but ventricular tissue, which accounted for most of the heart tissue contained within the ROI, generated the dominant frequency from the FFT, which we reported as heart rate. In the event of overlap with the atrium, the algorithm recorded only one contraction per cardiac cycle and did not register separate atrial and ventricular contractions. The algorithm is not currently equipped to measure arrhythmia, and the influence of arrhythmia on heart rate values reported here is unclear. Furthermore, the current iteration of the algorithm is designed to process only nd2-type video files and 11-bit images, as images degraded to 10 and 8 bits were not readable by the algorithm, although 11-bit images adjusted to approximate a slower sampling rate, were in fact readable by the algorithm. Importantly, the code may be adjustable to accommodate other file types. Finally, FisHRateZ, as currently constructed, is limited to assessment of heart rate in embryos prior to swim bladder inflation, and not older embryos. This was done to avoid technical challenges associated with anesthesia and restraint used to control mobility and the use of chemicals to limit pigmentation to maintain skin translucence.

Taken together, this automated video processing platform for the first time capitalized on zebrafish embryo shape and heart position to capture heart contraction patterns allowing high-throughput heart rate detection from multiple animals per imaging field without requirement for manual repositioning of animals. Paired with an intact model organism with relevant and translatable cardiac physiology and an easily measured endpoint, this approach may increase throughput of phenotype-based cardiotoxicity screening efforts of single compounds and mixtures in disciplines as disparate as ecology and drug discovery.

## Electronic supplementary material

Martin et al Supplementary Information

Supplementary Video_ Martin et al

Data S1

## Data Availability

FisHRateZ executable and code and Octave FFT code are available at: https://github.com/orgs/USEPA/teams/fishratez. Data used to generate figures are available as a supplementary spreadsheet.
