# Supplementary material for: High-Throughput Video Processing of Heart Rate Responses in Multiple Wild-type Embryonic Zebrafish per Imaging Field
Source: Sci Rep. 2019 Jan 15;9:145. doi: 10.1038/s41598-018-35949-5 (PMC6333808; doi:10.1038/s41598-018-35949-5)
Supplement: Supplementary file 1 — Martin et al Supplementary Information [file 41598_2018_35949_MOESM1_ESM.docx]

**Supplemental Information for “High-Throughput Video Processing of Heart Rate Responses in Multiple Wild-type Embryonic Zebrafish per Imaging Field”**

W. Kyle Martin^1^, Alan H. Tennant^2^, Rory B. Conolly^2^, Katya Prince^3^, Joey S. Stevens^4^, David M. DeMarini^2^, Brandi L. Martin^4^, Leslie C. Thompson^2^, M. Ian Gilmour^2^, Wayne E. Cascio^2^, Michael D. Hays^5^, Mehdi S. Hazari^2^, Stephanie Padilla^2^, Aimen K. Farraj ^2 *^

^1^Curriculum in Toxicology and Environmental Medicine, University of North Carolina at Chapel Hill, Chapel Hill, NC, USA ,^2^The National Health and Environmental Effects Research Laboratory, US EPA, RTP, NC, USA, ^3^ Prince Consulting, LLC, Durham, NC, USA, ^4^Oak Ridge Institute for Science and Education, Oak Ridge, TN, USA, ^5^National Risk Management Research Laboratory, US EPA, RTP, NC, USA

*CORRESPONDENCE:

Dr. Aimen K. Farraj, Ph.D., DABT

109 T.W. Alexander Dr

Mail Code: B105-02

Research Triangle Park, NC 27709

[p] 919-541-5027

[f] 919-541-0034

[e] [Farraj.aimen@epa.gov](mailto:Farraj.aimen@epa.gov)

| Exposure | Concentration |
| --- | --- |
| DMSO | 0.4% or 0.8% |
| Clonidine HCl | 3,10, 30, or 100 µg/ml |
| Epinephrine HCl | 10, 40, or 80 µg/ml |
| CDEP | 0.1, 1, 10, or 40 µg Extractable Organic Material/ml |
| B0 |  |
| B50 |  |
| B100 |  |
| Red Oak |  |

**Table S1.** **Zebrafish embryos were exposed to control drugs or organic extracts of air pollution-derived particulate matter.**


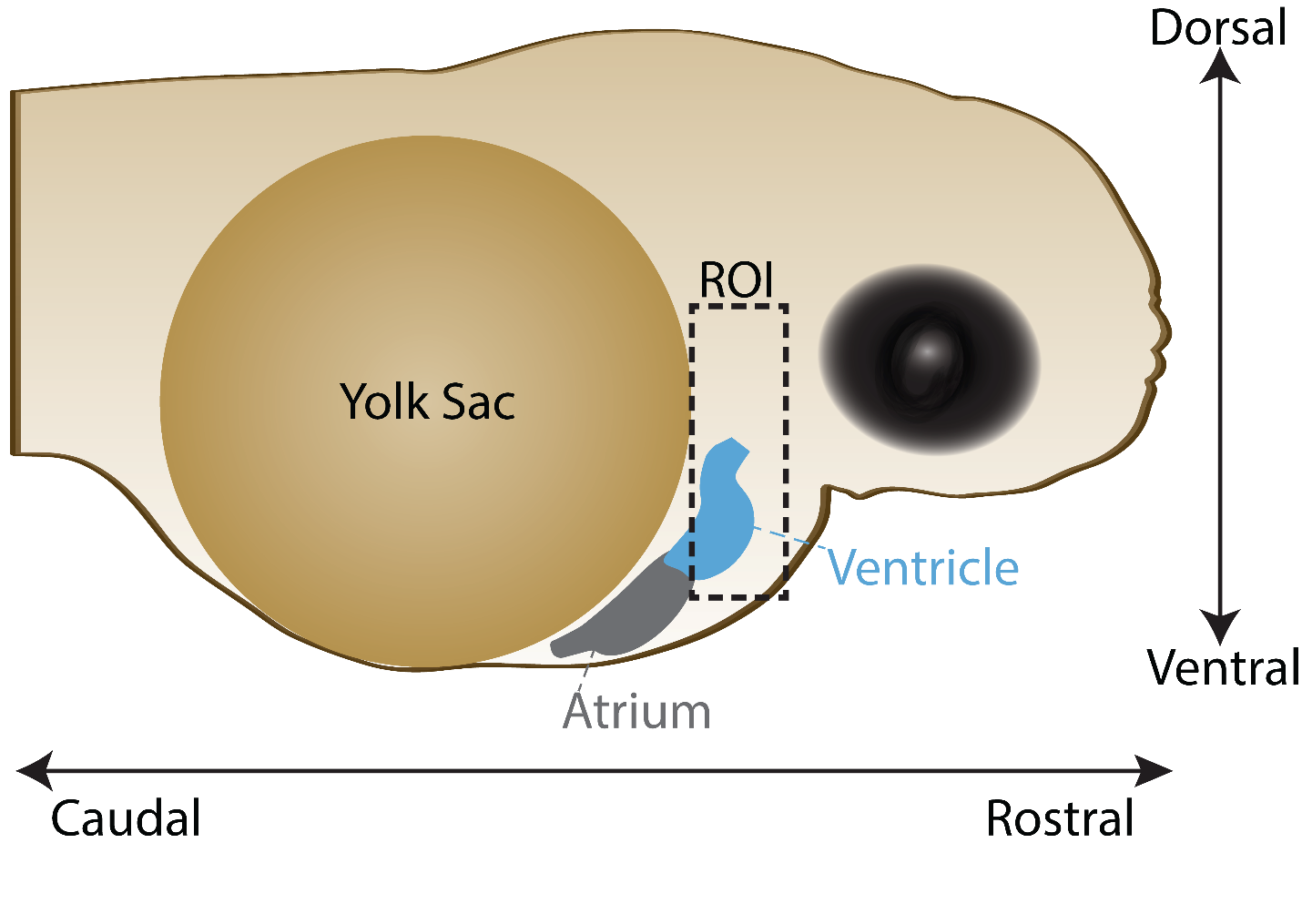


**Figure S1**. **FisHRateZ capitalizes on embryo shape to place Region of Interest box over heart.** ROI = region of interest


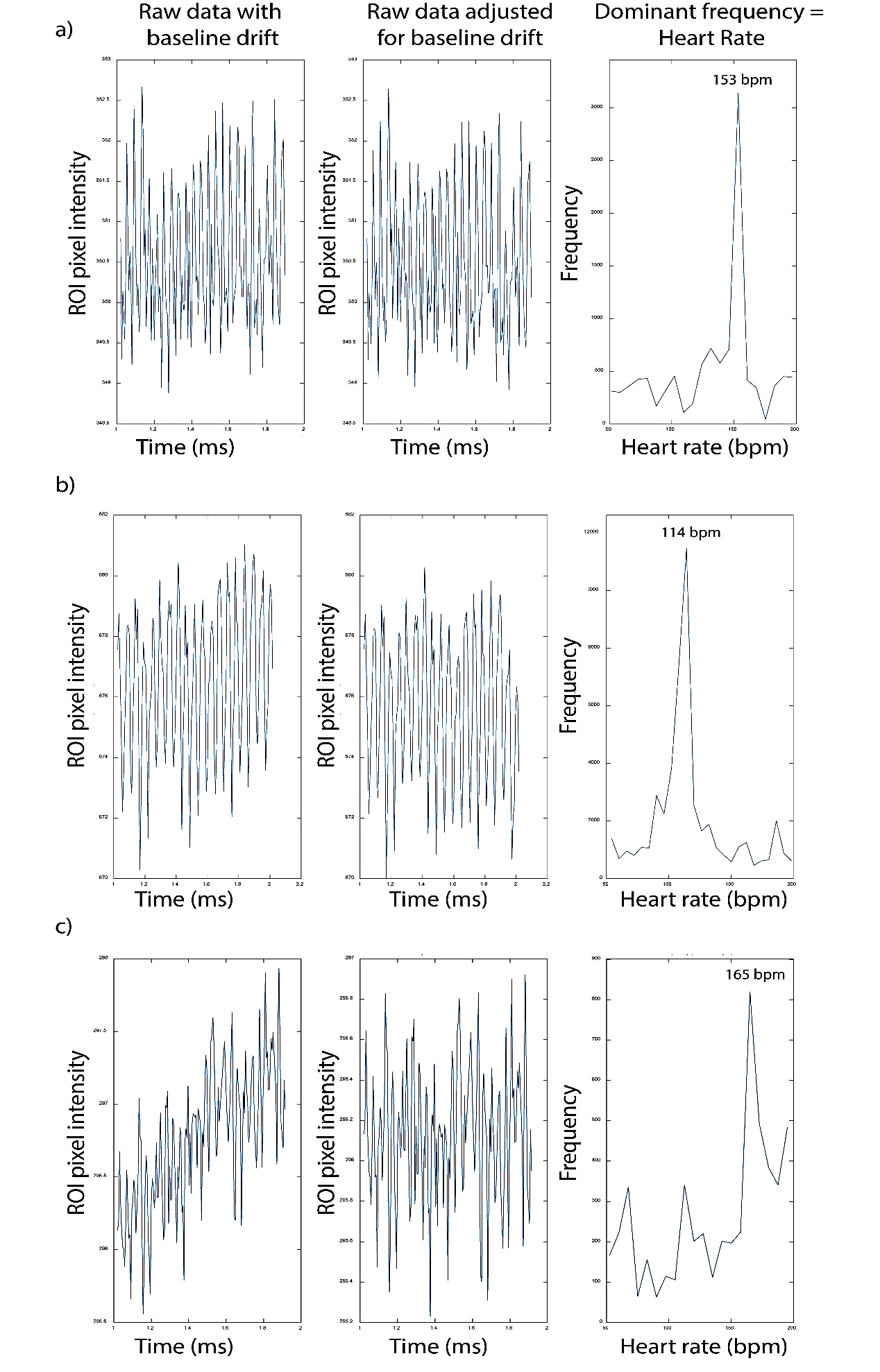


**Figure S2.** **A custom algorithm corrects for baseline drift and utilizes fast Fourier Transform to derive heart rate**. Representative uncorrected pixel intensity data (column one), baseline corrected data (column two), and fast Fourier-derived heart rate (column three) from a) DMSO-, b) Clonidine HCl-, or c) Epinephrine HCl-treated wild-type embryonic zebrafish.


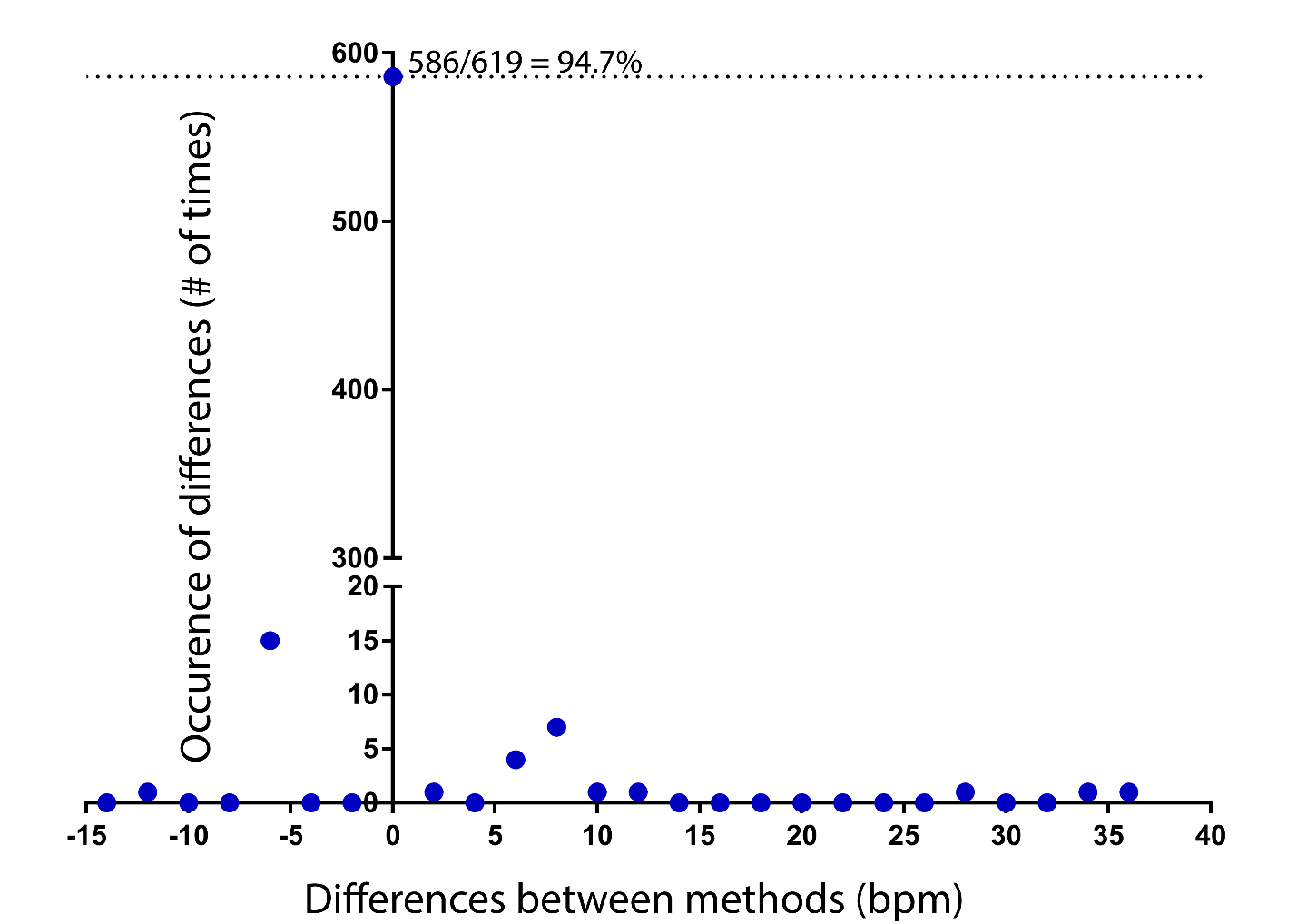


**Figure S3.** **Frequency distribution of the differences in heart rate between automated and manual placement scored using Bland-Altman methods comparison**. The x-axis represents the differences between automated and manual ROI placement-derived heart rates, and the y-axis represents the number of occurrences of the differences. Zero difference was scored in 94.7% of comparisons.


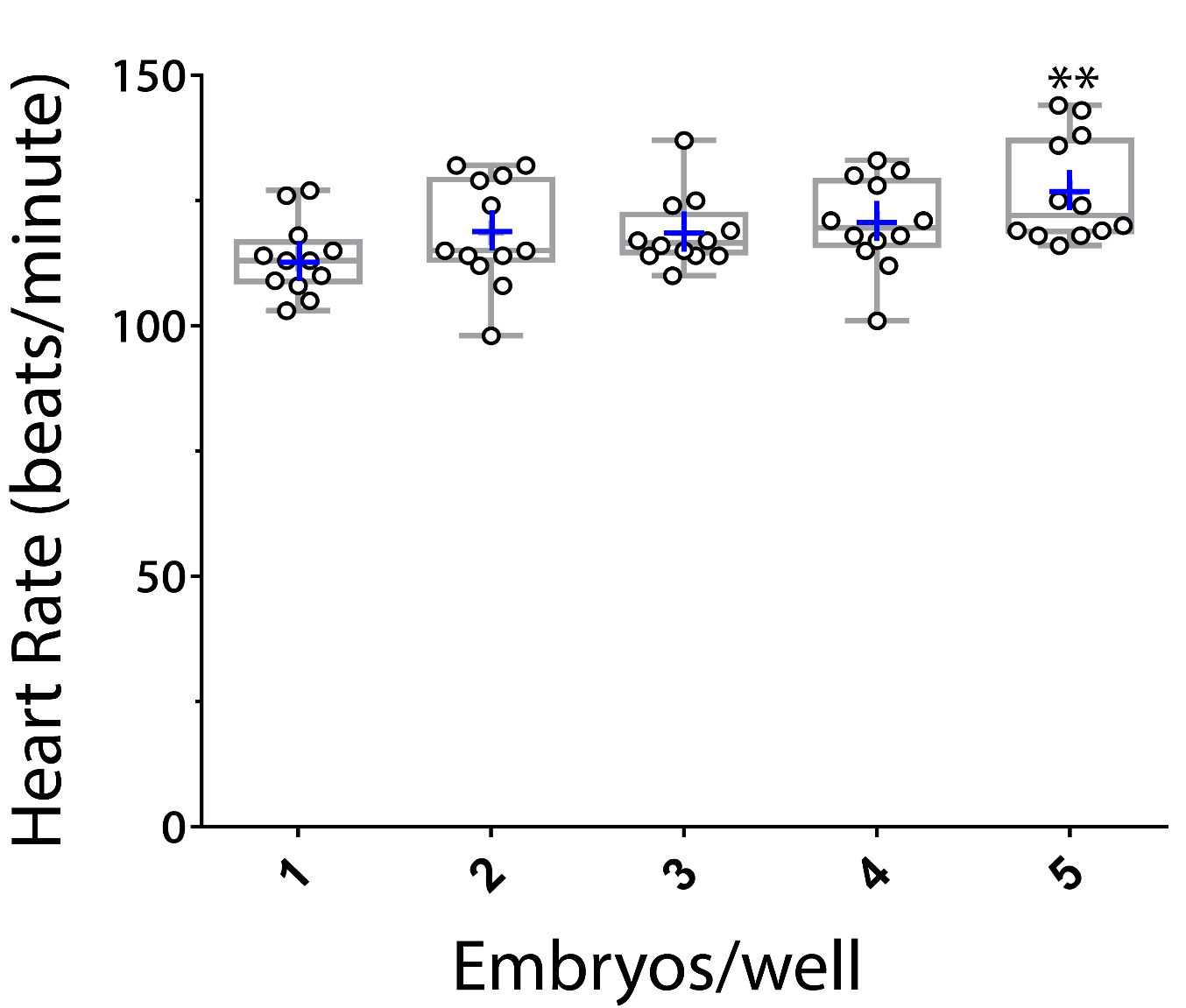


**Figure S4. Impact of co-housing on embryo heart rate.** One to five embryos were placed in wells and imaged as described in the Methods and Materials section. Single values indicate average heart rate per well with means of all wells per group (blue +) compared using one-way, two-tailed ANOVA and Tukey’s multiple comparisons test.

** p = 0.0068 vs. one embryo/well; n = 12/group. Boxplot description can be found in the Methods and Materials section.


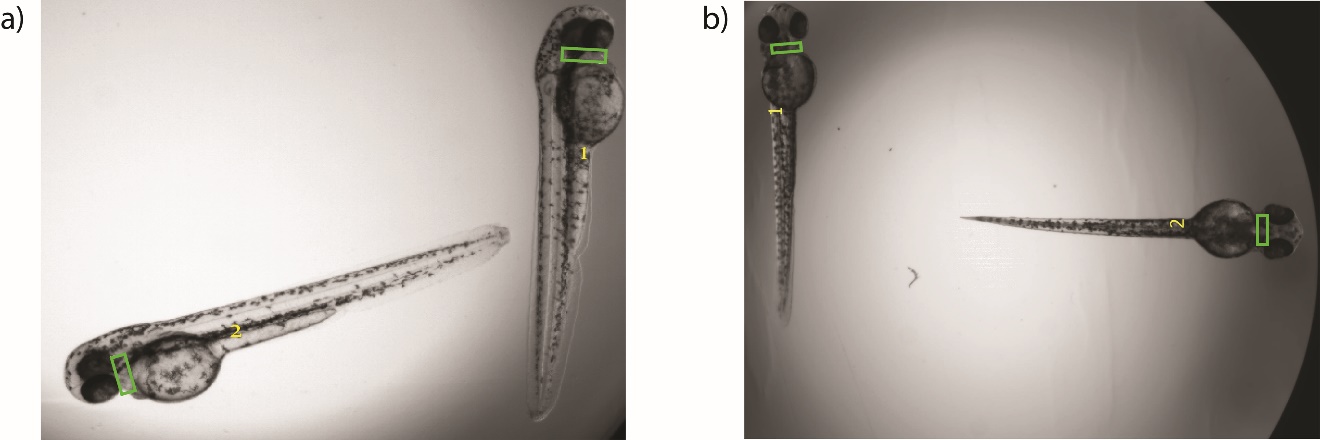


**Figure S5.** **FisHRateZ precisely placed Region of Interest boxes over the hearts of embryos that were positioned a) laterally or while b) supine (i.e., ventral surface of fish pointing upward).**


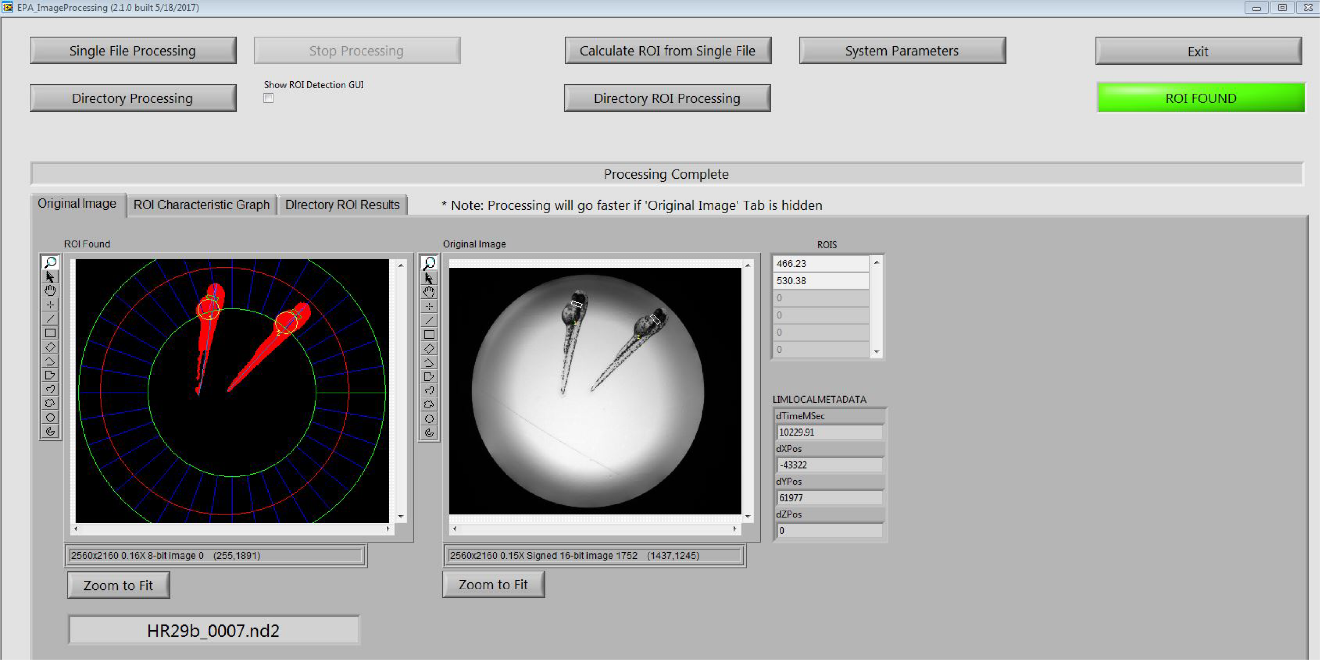


**Figure S6.** **Graphical user interface for FisHRateZ.**
